# Galectin-4 expression is down-regulated in response to autophagy during differentiation of rat trophoblast cells

**DOI:** 10.1038/srep32248

**Published:** 2016-08-30

**Authors:** Tomohiro Arikawa, Shengjun Liao, Hiroki Shimada, Tomoki Inoue, Hiromi Sakata-Haga, Takanori Nakamura, Toshihisa Hatta, Hiroki Shoji

**Affiliations:** 1 Department of Biology, Kanazawa Medical University, Uchinada, Ishikawa, Japan; 2 Medical Research Institute, Kanazawa Medical University, Uchinada, Ishikawa, Japan; 3 Department of Clinical Laboratory, Zhongnan Hospital of Wuhan University, Wuhan, Hubei, China; 4 Department of Anatomy, Kanazawa Medical University, Uchinada, Ishikawa, Japan; 5 Department of Mathematics, Kanazawa Medical University, Uchinada, Ishikawa, Japan; 6 Department of Endocrinology, Faculty of Medicine, Kagawa University, Kagawa, Japan

## Abstract

Placental development and trophoblast invasion of the maternal endometrium establish the maternal-fetal interface, which is critical for the developing embryo and fetus. Herein we show that overexpression of Galectin-4 (Gal-4) during trophoblast differentiation inhibited the enlargement of Rcho-1 cells (a model for rat trophoblast differentiation) and promoted cell-cell adhesion, whereas trophoblast specific markers and MMP-9 activity were not affected. In the rat placenta, microtubule associated protein 1 light chain 3 alpha (LC3) protein, an autophagy marker, is highly expressed on the maternal side of the decidua where Gal-4 expression is weak. *In vitro* assays showed that the expression of trophoblast-specific differentiation markers was reduced by 3-Methyladenine (3-MA) and Bafilomycin A1, known as autophagy inhibitors, compared to control cells. Furthermore, Gal-4 expression in Rcho-1 cells, which is normally down-regulated during differentiation, was not attenuated in the presence of autophagy inhibitors, suggesting that autophagy is upstream of Gal-4 expression. We herein describe a possible mechanism by which autophagy regulates trophoblast differentiation *via* regulation of Gal-4 expression in order to establish the maternal-fetal interface.

Trophoblasts, which originate from the marginal zone of the blastocyst, are abundant cells in the placenta and influence both fetal and placental development by infiltrating the maternal endometrium during early implantation^1^. This infiltration by trophoblasts is crucial for the establishment of the maternal-fetal interface^2^,^3^. It has been determined that the invasive ability of trophoblasts is regulated by various environmental factors, including signaling by adhesion molecules and growth factors, regulated by the interactions of the decidua and trophoblasts in the endometrium. Autophagy is a self-degradative process that is pivotal for balancing sources of energy during development and in response to nutrient/oxygen stresses^4^,^5^; this catabolic process involves the bulk degradation of cytoplasmic components for cellular homeostasis. Nakashima *et al*. showed that autophagy is essential for the invasion of extravillous trophoblasts (EVTs) and for EVT-vascular remodeling under physiological hypoxia during placentation^6^.

Galectins are a family of animal lectins which recognize β-galactoside-containing carbohydrate moieties. They have been proposed to regulate a wide variety of biological processes such as immunity, cell differentiation, cell adhesion, cancer growth and metastasis^7^. To date, it has been reported that Gals-1, -3, -8, -9, -13, and/or -17 are involved in placentation, maternal immune tolerance, and disorders of pregnancy, such as preeclampsia^8^,^9^,^10^,^11^. Among them, some galectins such as Gals-1, -8 and -9 have shown to be implicated in autophagy; Gal-1 has been shown to induce autophagy in hepatocellular carcinoma, which leads to chemoresistance^12^. Gal-8 has been shown to play a role in Listeria uptake through the formation of autophagosomes^13^. On the other hand, Gal-9 has been shown to negatively regulate autophagy in KRAS mutant colon carcinoma, which results in cell death^14^. Given the nutrient-poor conditions that exist during early placentation as blood vessels have not yet developed, it is possible that galectins are involved in placentation in association with autophagy.

In addition, we have recently reported that Galectin-4 (Gal-4), which is known to be dominantly expressed in the digestive tract^15^, is also expressed at the maternal-fetal interface during rat placentation^16^. We further showed that the expression of Gal-4 was down-regulated during differentiation of rat trophoblast-derived Rcho-1 cells^16^, a good model system for rat trophoblast differentiation. The expression of Gal-4, however, had only been analyzed in bovine epitheliochorial placenta^17^, but not in hemochorial placenta. The roles identified for Gal-4 in previous studies included immune modulation^18^, polarized membrane trafficking^19^, lipid raft stabilization^20^, wound healing^21^, and cancer cell invasion^22^,^23^. Interestingly, previous reports have shown that Gal-4 inhibits cancer metastasis^24^,^25^. Therefore, it was important to determine if Gal-4 is involved in the differentiation and invasion of rat Rcho-1 cells.

Previous works showed that differentiation of Rcho-1 cells can be induced by exchanging the culture medium from RPMI1640 containing 20% bovine serum to NCTC medium containing 1% horse serum^16^. We thus hypothesized that low-nutrient condition could allow Rcho-1 cells to induce autophagy. In this study, we show that LC3 and Gal-4 have complementary expression in the placenta, therefore we also investigated the involvement of Gal-4 in autophagy-mediated placentation. We show that down-regulation of Gal-4 in trophoblasts is critical for their ability to migrate into the endometrium during autophagy-mediated placentation.

## Results

### Differentiation of rat Rcho-1 cells and expression of Gal-4

**Figure 1:**
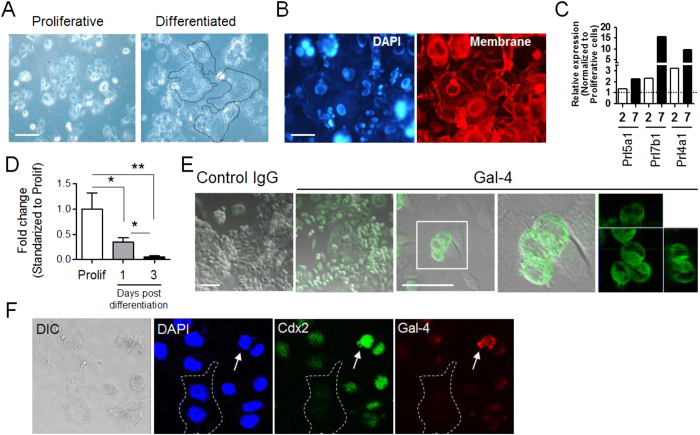
Changes in morphology and differentiation-specific markers of Rcho-1 cells and expression pattern of Gal-4 during Rcho-1 cell differentiation. (**A**) Typical shape of Rcho-1 cells during proliferation and after differentiation. The dotted line represents typical enlarged Rcho-1 cells in comparison with proliferation phase cells. Proliferative: proliferation phase, differentiated: Differentiated phase. The scale bar represents 30 μm. (**B**) Cell membrane staining. The scale bar represents 30 μm. (**C**) Expression of trophoblast-specific genes was analyzed using microarray analysis of total RNA from Rcho-1 cells harvested on day 2 and day 7 post-differentiation as described in the Materials and Methods. The dotted line indicates the pre-differentiation expression level of each gene. (**D**) *Gal-4* expression was down-regulated on day1, and day3 post-differentiation in Rcho-1 cells. *P < 0.05, **P < 0.01. Prolif: proliferative cells. (**E**,**F**) Immunocytochemical analysis of the distribution of endogenous Gal-4 protein in proliferative Rcho-1 cells (**E**) and co-localization of Cdx2 and Gal-4 in early differentiation phase (**F**: Day 1 post differentiation). Cytoplasmic localization of Gal-4 protein and nucleic localization of Cdx2 in same cells was observed with confocal microscopy. Dotted line represents enlarged Rcho-1 cells. Arrows indicate Rcho-1 cell which expresses both Gal-4 and Cdx2. The scale bar represents 30 μm.

Rcho-1 cells were differentiated with the low-nutrient medium, and enlarged Rcho-1 cells which have large nuclei were observed during differentiation (Fig. 1A,B). We examined the expression of mRNA for differentiation-specific markers using a DNA microarray assay. Of note, *Prl5a1, Prl7b1,* and *Prl4a1* mRNA known as specific markers for invasive trophoblasts were up-regulated during differentiation of Rcho-1 cells^26^,^27^ (Fig. 1C). These results suggested that Rcho-1 cells are mainly capable of differentiating into invasive trophoblasts and trophoblast giant cells, consistent with published reports^28^. We have previously shown that *Gal-4* is down-regulated in post-differentiated Rcho-1 cells (Fig. 1D)^16^. When we analyzed the expression of Gal-4 protein in growth phase Rcho-1 cells cultured in nutrient-rich medium, Gal-4 localized to the cytoplasm of rounded cells, but not enlarged cells (Fig. 1E). These enlarged cells are likely to be differentiated cells which naturally formed a small population. We thus attempted to assess whether Gal-4 expression is observed in undifferentiated Rcho-1 cells with immunocytochemical staining for Cdx2, known as stem cell marker (Fig. 1F). We observed strong signal of Gal-4 in rather small cells where Cdx2 signal was also strong. And there were no significant signal of both Gal-4 and Cdx2 in large cells, indicating that Gal-4 is expressed in undifferentiated Rcho-1 cells. Also, these observations suggested that *Gal-4* down-regulation may be involved in placentation. We thus assessed the role of Gal-4 in Rcho-1 cell differentiation *in vitro*.

### Rescue of Gal-4 expression during trophoblast differentiation inhibits the enlargement of Rcho-1 cells and promotes cell-cell adhesion

**Figure 2:**
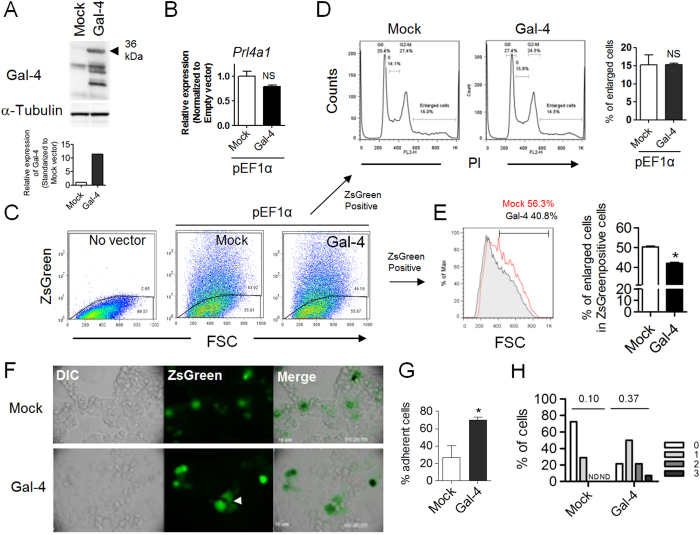
Gal-4 overexpression during Rcho-1 differentiation interferes with trophoblast enlargement and cell mobility. (**A**) Western blot assay of lysates from Rcho-1 cells at 48 hrs after Gal-4 overexpression as described in the Materials and Methods. Full-length blot is shown in Supplementary Fig. S1. (**B**) Expression of *Prl4a1* in differentiated Rcho-1 cells (7 days after induction of differentiation) was analyzed by Real-time RT-PCR. Gal-4 overexpression did not affect the level of *Prl4a* mRNA. NS: not significant. (**C**,**E**) The effect of Gal-4 overexpression on the enlargement of Rcho-1 cells which occurs during differentiation was analyzed by flow cytometry. The proportion of larger cells with higher forward scatter (FSC) among ZsGreen positive cells (plasmid-incorporated cells) was decreased in Gal-4 overexpressing cells compared to cells transfected with an empty vector. The proportion of large cells was compared by histogram (**E**). *p < 0.05. (**D**) The impact of Gal-4 overexpression on the ploidy of Rcho-1 cells. The ploidy of Rcho-1 cells was not affected by Gal-4 overexpression. NS; not significant. (**F**) Representative image of Rcho-1 cells transfected with empty or *Gal-4* cDNA vectors at the end of analysis (60 hours post-transfection). Gal-4 overexpressing cells form more contacts with each other than ZsGreen-positive control cells (Arrow head). Phase contrast (DIC) and fluorescence images (Green) were merged. (**G**) The proportion of ZsGreen-positive cells in contact with other ZsGreen-positive cells was compared in the histogram. Two days after transfection, we selected 18 or 23 ZsGreen-positive cells which had no contact with other ZsGreen-positive cells from the control and Gal-4 overexpressing cells, respectively. After 12 hours, the positioning of each selected cell was analyzed to determine if it was in contact with other ZsGreen-positive cells, if so, we counted the number of adhered cells. *P < 0.05. (**H**) The proportion of cells in contact with 0–3 ZsGreen-positive cells is shown in the histogram. The proportion per unit time of adherent cells (*p*) shown in the histogram was calculated by a least squares method as described in the Materials and Methods.

To clarify the role of Gal-4 in Rcho-1 cell differentiation, Gal-4 was overexpressed during Rcho-1 cell differentiation using the pEF1α plasmid, in which full-length Gal-4 has been inserted as described in the Materials and Methods. By Western blot assay, the expected protein comprised the main band at 36 kDa (Fig. 2A). The smaller proteins were likely products of proteolysis, since the linker peptide of tandem-repeat-type galectin is highly susceptible to proteolysis. At first, we attempted whether Gal-4 overexpression affects on Rcho-1 differentiation with monitoring the *Prl4a1* expression. *Prl4a1* expression was not affected with Gal-4 overexpression (Fig. 2B). Next, we tried to explore the impact of Gal-4 overexpression on the ploidy and cell morphology of Rcho-1 cells. The efficacy of *Gal-4* cDNA transfection in Rcho-1 cells was monitored by ZsGreen fluorescent protein whose cDNA was tandemly introduced into the vector with *Gal-4* cDNA (Fig. 2C). Gal-4 overexpressing cells were induced to differentiate, and then the ploidy and the size distribution of cells was analyzed with a Flowcytometric assay. Results showed no effect on the ploidy, but a decrease in the ratio of Gal-4-overexpressing enlarged cells compared to cells transfected with mock vector (Fig. 2D,E), indicating that overexpression of Gal-4 suppressed the enlargement of Rcho-1 cells, but not affect on DNA content in nuclei during Rcho-1 differentiation.

In the next experiment, we traced the behavior of ZsGreen-positive cells. Two days after transfection, we selected 18 or 23 ZsGreen-positive cells which had no contact with other ZsGreen-positive cells, and observed them with time-lapse microscopy (Fig. 2F). After 12 hours we analyzed the position of each selected cell to determine if it was in contact with other ZsGreen-positive cells; if it was, we counted the number of cells to which it was adhered (Fig. 2F). As shown in Fig. 2G, the ratio of ZsGreen-positive cells in contact with other ZsGreen-positive cells was significantly higher in Gal-4-overexpressing cells than in control cells. Furthermore, when Gal-4 was overexpressed, cells were found to be in contact with two or three cells, while contact with only one cell or none was observed among control cells (Fig. 2H). By means of a least squares method, we calculated the probability (*p*) of adhesion with ZsGreen-positive cells in Gal-4-overexpressing cells and controls. The probability of adhesive ZsGreen cells among the Gal-4-overexpressing cells was *p* = 0.37; in contrast that of controls was *p* = 0.10. These results demonstrated that Gal-4-positive cells tended to adhere in a homophilic manner compared to cells transfected with mock vector. Taken together, our results suggest that Rcho-1 cells down-regulate Gal-4 expression during differentiation to gain invasive ability.

In the next experiment, we examined the possibility that Gal-4 plays a role in placentation in connection with autophagy, as it has been recently shown that autophagy is involved in placentation. Moreover, it has been shown that some galectins are involved in the regulation of autophagy^12^,^13^,^14^.

### Gal-4 mainly localizes to fetal tissue, while LC3 protein is strongly detected on the maternal side

**Figure 3:**
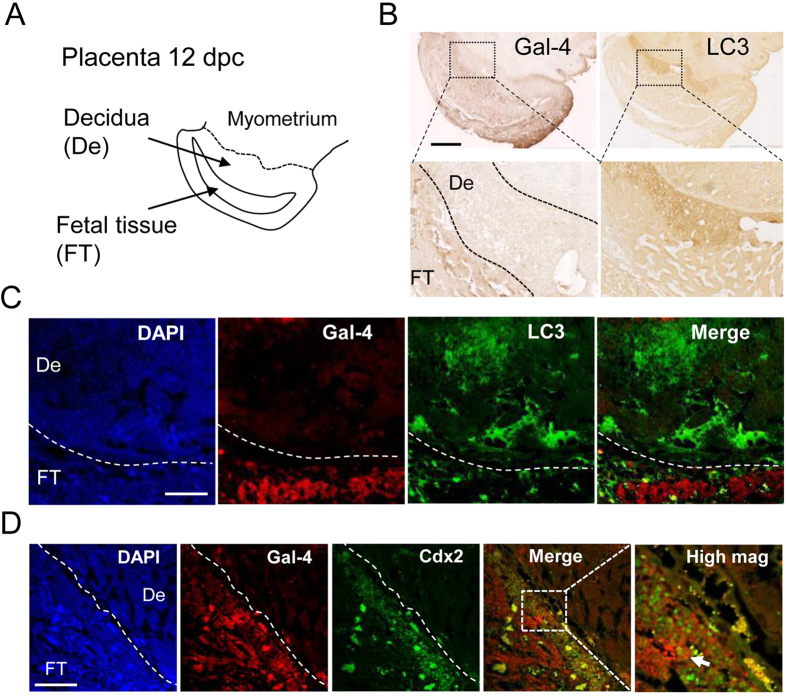
Immunohistochemical assays for LC3 and Gal-4 in 12 dpc rat placenta. (**A**) Diagram of the 12 dpc rat placenta. (**B**) Gal-4 protein is not abundant on the maternal side of the decidua where LC3 is strongly expressed. Typical data from three independent experiments are shown. The scale bar represents 1 mm. (**C**) Fluorescence immunohistochemistry for Gal-4 and LC3 in the 12 dpc rat placenta. The scale bar represents 100 μm. (**D**) Fluorescence immunohistochemistry for Gal-4 and Cdx2 in the 12 dpc rat placenta. Arrow indicates co-localization of Gal-4 and Cdx2 in same cell. The scale bar represents 30 μm.

Immunohistochemistry (IHC) assays showed that Gal-4 was expressed in fetal tissue, while LC3 protein was strongly detected on the maternal side of the decidua (Fig. 3B,C). Therefore, the distribution of Gal-4 and LC3 seemed to be complementary. Fluorescence confocal microscopy assay showed that the expression of Cdx2, which is a homeodomain protein expressed in the stem cells, was coincided with Gal-4 in the fetal tissue (Fig. 3D), indicating that stem cells in the placenta express Gal-4.

**Figure 4:**
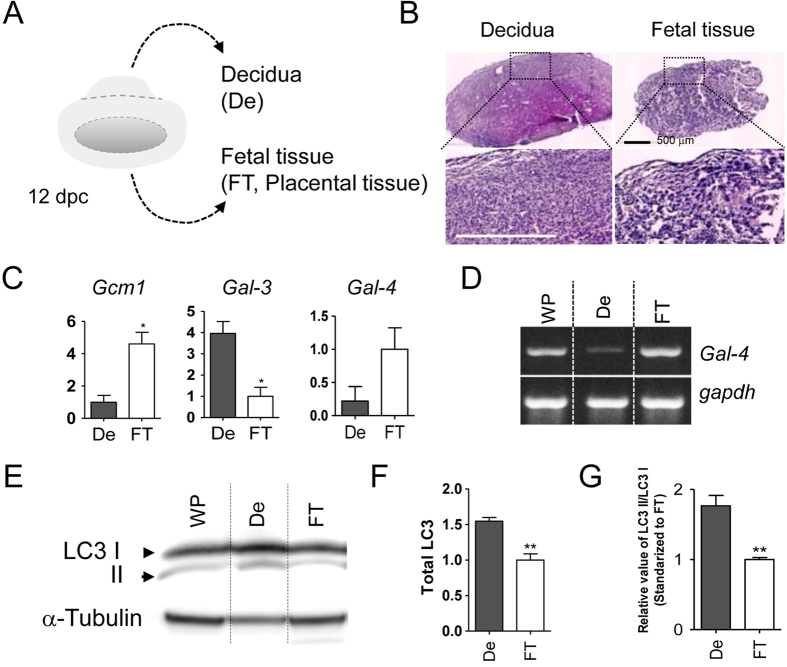
LC3 and Gal-4 show complementary expression in rat placenta. (**A**) Schematic diagram of the removal of the decidua and fetal tissue from a 12 dpc rat placenta. (**B**) Hematoxylin and eosin staining of harvested fetal and decidual tissue. The scale bar represents 500 μm. (**C**) Quantitative RT-PCR for *Gcm1*, *Gal-3*, and *Gal-4* in fetal tissue (FT) and decidua (DE). *P < 0.05. (**D**) Expression pattern of Gal-4 in whole placenta (WP), decidual and fetal tissue by conventional RT-PCR. (**E**) Western blot analysis for LC3 was performed with cell lysates obtained from whole placenta, decidual and fetal tissue. The graph represents total LC3 concentration (**F**) and the LC3II/ LC3I ratio (**G**). *P < 0.05, **P < 0.01. Full-length gel and blot are shown in Supplementary Fig. S2.

We next physically separated the decidua and the fetal tissue from a rat 12 days post-coitum (dpc) rat placenta, then prepared cell lysates for Western blot analysis and collection of total RNA for quantitative RT-PCR. Figure 4A diagrams a 12 dpc placenta and Fig. 4B shows the separated specimens with HE staining. We confirmed that the maternal decidua and fetal tissue were comprised of rounded cells with a high density and tissue containing trophoblast giant cells, respectively. Furthermore, we detected the presence of marker genes of fetal tissue, *Gcm1* and *galectin-3* which are highly expressed in the decidua, as expected by quantitative RT-PCR (Fig. 4C). Consistent with the IHC results, mRNA for Gal-4 was found to be mainly expressed in fetal tissue (Fig. 4C,D). As the changing of LC3 protein form, shifting from LC3I to LC3II, is a well-known marker of autophagy^14^, we further assessed LC3 protein by Western blot assay. Figure 4E showed that both the total amount of LC3 and the ratio of LC3II/I (LC3II/LC3I) were higher in the decidua than in the fetal tissue, suggesting that autophagy plays a role in establishing the maternal-fetal interface. Of interest, Gal-4 expression was much weaker on the maternal side of the decidua where autophagy seemed to be accelerated (Fig. 4D). Thus, this complementary expression pattern, together with the observation that Gal-4 expression is down-regulated during Rcho-1 cell differentiation, led us to hypothesize that the diminishment of Gal-4 expression by autophagy regulates trophoblast adhesion.

### 3-methyladenine (3-MA) and Bafilomycin A1, autophagy inhibitors, suppress the down-regulation of Gal-4 expression during Rcho-1 differentiation

**Figure 5:**
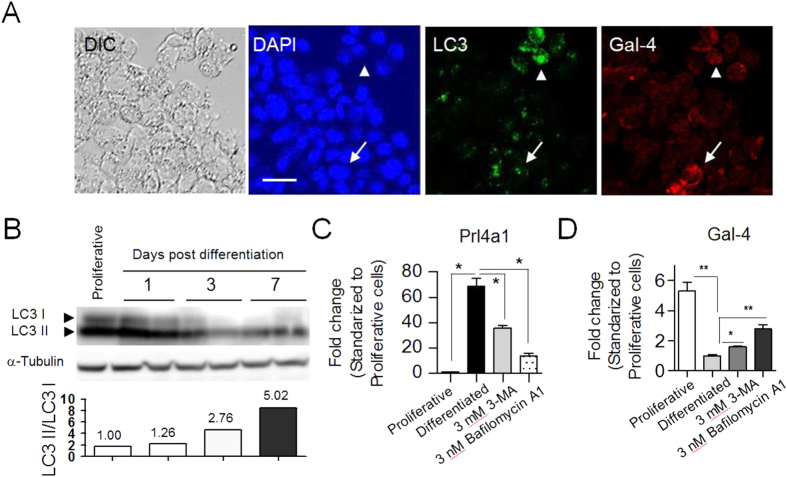
Autophagy regulates differentiation of Rcho-1 cells. (**A**) Immunocytochemical assay for LC3 and Gal-4. Arrows indicate Gal-4 expressed Rcho-1 cells. Arrowheads indicate cells with LC3 puncta in cytoplasmic region. The scale bar represents 10 μm. (**B**) Western blot showing the increased ratio of LC3 II during Rcho-1 cell differentiation. Three independently cultured cell populations from each phase (proliferative state, 0, 2 and 7 days after induction of differentiation) were analyzed. The ratio of LC3 II is shown as a relative value standardized to the proliferative cells in the histogram. Proliferative: proliferative phase. Full-length blot is shown in Supplementary Fig. S3. (**C**) Real-time RT-PCR analysis of expression of *Prl4a1*, a trophoblast differentiation-specific gene, during differentiation of Rcho-1 cells with or without 3 mM of 3-MA or 3nM of Bafilomycin A1. The mRNA level is shown as a relative value standardized to proliferative cells. Expression of *Prl4a1* was inhibited by 3-MA and Bafilomycin A1. *P < 0.05. (**D**) Real-time RT-PCR analysis of *Gal-4* expression during differentiation of Rcho-1 cells with or without 3-MA or Bafilomycin A1. The mRNA level is shown as a relative value standardized to proliferative cells. 3-MA and Bafilomycin A1 prevent the down-regulation of Gal-4 expression in response to Rcho-1 cell differentiation. *P < 0.05, **P < 0.01.

Next, we sought to determine if autophagy also occurs during differentiation of rat Rcho-1 cells. Immunocytochemical assay in early differentiation phase (Day 1 post differentiation) showed that Rcho-1 cells with LC3 puncta did not express Gal-4 (arrow head in Fig. 5A). In addition to that, those with Gal-4 do not have LC3 puncta (arrow in Fig. 5A). Western blot assays showed that the LC3II/I ratio gradually increased (Fig. 5B), indicating the possibility that autophagy is involved in differentiation of rat Rcho-1 cells. We thus tried to inhibit autophagy using 3-MA and Bafilomycin A1, which are known as general inhibitors of autophagy. In general, Bafilomycin A1 is used for cell culture at 100 nM as a working concentration. However, since Bafilomycin A1 is supposed to be toxic and Rcho-1 differentiation system requires longer period (one week) than the general procedures (a few hours culture), we determined the optimal concentration of Bafilomycin A1 in our study. As a result, the maximal concentration was determined at 3 nM (Data not shown). On the other hand, it has been shown that concentrations of DMSO greater than 0.5% affect the differentiation of trophoblasts^29^; we therefore used DMSO as a solvent for 3-MA at a final concentration of less than 0.5%. Addition of either 3 mM 3-MA or 3 nM Bafilomycin A1 in the differentiation medium suppressed the up-regulation of trophoblast-specific marker, *Prl4a1,* expression indicating that autophagy may promote trophoblast differentiation (Fig. 5C). We next assessed whether Gal-4 expression is regulated by autophagy. Rcho-1 cells were allowed to differentiate in medium containing either 3 mM 3-MA or 3 nM Bafilomycin A1, and quantitative RT-PCR was performed using total RNA of cells harvested 3 days after the induction of differentiation. As shown in Fig. 5D, mRNA levels were higher in cells cultured with either 3-MA or Bafilomycin A1 than in control cells, indicating that inhibition of autophagy suppressed the down-regulation of *Gal-4* expression. This result suggests that autophagy down-regulates Gal-4 expression during Rcho-1 cell differentiation.

### Inhibition of autophagy suppresses the up-regulation of MMP activity during the differentiation of Rcho-1 cells

**Figure 6:**
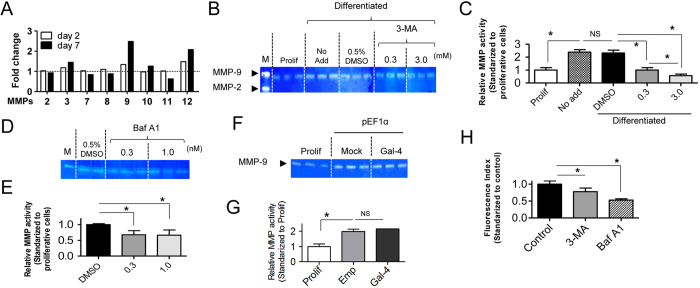
Suppressive effect of autophagy inhibitors on differentiation of Rcho-1 cells and MMP activity. (**A**) DNA microarray analysis of MMP expression during Rcho-1 cell differentiation. The levels of MMP mRNA from cells at 2 and 7 days after induction of differentiation are shown as relative values standardized to proliferative cells. (**B**) MMP-9 activity was analyzed by Zymography assay which showed up-regulation of activity after differentiation in control cells, while the up-regulation was suppressed by the addition of 3-MA in a dose dependent manner (0.3–3 mM of 3-MA). Three independently cultured cell populations were analyzed for each condition. (**C**) The activity of MMP-9 in B is shown as a relative value standardized to proliferative cells. *P < 0.05. Prolif; proliferative cells. (**D**) MMP-9 activity of Bafilomycin A1-treated Rcho-1 cells was analyzed with Zymography assay. (**E**) The activity of MMP-9 in D is shown as a relative value standardized to proliferative cells. *P < 0.05. Prolif; proliferative cells. (**F**) MMP-9 activity of Gal-4-overexpressed Rcho-1 cells was analyzed with Zymography assay. The activity was upregulated at 7 days after induction of differentiation in both control (transfected empty vector) and Gal-4 overexpressing cells. Three independently cultured cell populations were analyzed for each condition. (**G**) MMP-9 activity in F is shown as a relative value standardized to proliferative cells. *P < 0.05. NS: Not significant. (**H**) Effect of 3 mM 3-MA and 3 nM Bafilomycin A1 on invasive activity of differentiated Rcho-1 cells was analyzed with invasion assay. *P < 0.05. Full-length gels are shown in Supplementary Fig. S4.

It is thought that invasive trophoblasts invade maternal tissue using matrix metalloproteinases (MMPs)^30^, therefore we examined MMP expression during Rcho-1 differentiation by DNA microarray, and the effects of 3-MA. Consistent with previous reports^30^, we observed enhanced MMP-9 expression in Rcho-1 cells after differentiation into invasive trophoblasts (Fig. 6A). Furthermore, the activity of MMP-9 was shown to be up-regulated during differentiation using a Zymography assay. Of note, this up-regulation was suppressed by the addition of either 3-MA or Bafilomycin A1 (Fig. 6B–E). We also attempted to assess the involvement of Gal-4 in the regulation of MMP activity (Fig. 6F,G) because Gal-4 overexpression affected enlargement and cell-cell adhesion of Rcho-1 cells (Fig. 2). However, Gal-4 overexpression affected neither the expression of *Prl4a1*, a trophoblast-specific marker, nor MMP-9 activity, indicating that Gal-4 is partially involved in regulating the differentiation process in Rcho-1 cells. We thus attempted to assess the effect of autophagy inhibitors using invasion assay (Fig. 6H). Inhibition of autophagy resulted in suppression of invasive activity of Rcho-1 cells, indicating that autophagy facilitates Rcho-1-cells invasion.

## Discussion

Here we demonstrate the involvement of autophagy and regulation of Galectin-4 (Gal-4) expression in the differentiation process of the rat Rcho-1 trophoblast cell line. Gal-4 expression is down-regulated during the early steps of Rcho-1 differentiation^16^. The results of the present study suggest that this down-regulation of Gal-4 expression is triggered by autophagy, demonstrated by the ability of the autophagy inhibitors, 3-MA and Bafilomycin A1, to abolish this down-regulation. We further assessed the effects of inducing differentiation in Gal-4 over-expressing Rcho-1 cells. Results showed that overexpression of Gal-4 leads to changes in cell mobility *via* homophilic cell-cell contact in Gal-4-positive cells. As previous work has shown that galectins tend to aggregate cells with glycoproteins expressed on the cell surface^31^,^32^, our observed results are consistent with the inherent functions of the galectin family. We also observed that the cellular enlargement which occurs during Rcho-1 cell differentiation was suppressed compared to control cells. Gal-4 has been reported to be strongly expressed in epithelial cells of the digestive tract^15^ and is involved in the apical proteins and membrane trafficking of epithelial cells^19^. We thus hypothesize that down-regulation of Gal-4 is necessary to alter the epithelial character which leads to the dissociation of cell-cell interactions allowing cells to acquire an invasive character and grow larger in size. However, a surplus of Gal-4 does not seem to interfere with further differentiation as overexpression of gal-4 does not affect differentiation-specific gene expression or up-regulation of MMP activity. Nevertheless, the complementary expression pattern of LC3 and gal-4 in the rat placenta led us to conclude that autophagy-mediated down-regulation of Gal-4 may be pivotal in placentation. The role of Gal-4 in trophoblast differentiation might also occur in some cancer systems, such as pancreatic adenocarcinoma^24^,^25^ and colorectal cancer^22^, where down-regulation of gal-4 is highly associated with the acquisition of invasive phenotypes. Similar mechanism as in our system has been proposed for inhibition of pancreatic adenocarcinoma migration by Gal-4, where Gal-4 has been suggested to act as an adhesion molecule to prevent release of the tumor cells^24^.

Autophagy is a well-known cellular mechanism involved in regulating differentiation and protein clearance, among others^4^,^5^. Because trophoblast exposure to the maternal circulation is restricted during early stages, it is thought that nutrients are not fully provided to the fetus during early stages. Trophoblasts would be exposed to hypoxic and low-nutrient conditions. Both hypoxia and low-nutrient concentrations can induce autophagy, and autophagy is observed in human placentation. Although previous studies showed that hypoxia inhibits trophoblast differentiation^33^,^34^, Rosario *et al*.^35^ and Nakashima *et al*.^6^ indicated the possibility of which hypoxia activates trophoblast invasion to the maternal side. Of note, Nakashima *et al*. showed that physiological hypoxia induces HIF1 expression resulting in autophagy activation and subsequent invasion of human extravillous trophoblasts into maternal tissue^6^. In this study, we have shown that autophagy may also be involved in rat placentation. The ratio of LC3II/LC3I was higher on the maternal side of the decidua than in fetal tissue, suggesting that autophagy is predominantly induced in the decidua. Moreover, *in vitro* assays showed that differentiation of Rcho-1 cells into invasive trophoblasts was suppressed by inhibition of autophagy with 3-MA and Bafilomycin A1, suggesting that autophagy is crucial to rat placentation.

Since recent work has shown that another member of the galectin family, Gal-8, is required for autophagosome formation^13^, we assessed the involvement of Gal-4 in autophagy regulation during the differentiation of Rcho-1 cells. Results indicated that autophagy was up-stream of the regulation of Gal-4 expression, whereas down-regulation of Gal-4 expression was suppressed by inhibiting autophagy. Therefore, it is suggested that autophagy triggers both down-regulation of Gal-4, which may free Rcho-1 cells from an epithelial state, and also promotes the differentiation into invasive cells.

**Figure 7:**
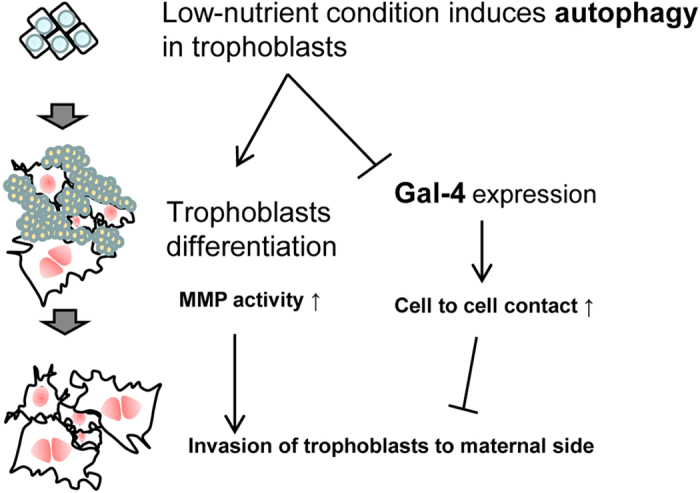
A schematic diagram of rat trophoblast differentiation in the current study.

In conclusion, we propose a novel mechanism by which autophagy positively regulates rat placentation and Gal-4 modulates fetal trophoblast invasion into maternal tissue to establish the maternal-fetal interface (Fig. 7). Autophagy has been shown to be associated with preeclampsia^6^. It would thus be interesting to determine if Gal-4 also shows an association with preeclampsia, and if both autophagy and Gal-4 are related to placenta accreta, which is characterized by the excessive invasion of trophoblasts^36^. These findings open new areas of research wherein the down-regulation of Gal-4 is crucial for the promotion of trophoblast cell invasion.

## Methods

### Cell culture

Rcho-1 cells were kindly provided by Professor Michael J. Soares (Department of Physiology, University of Kansas Medical Center, Kansas City, KS). Protocols for the maintenance and differentiation of Rcho-1 cells were in accordance with previous work^16^. The cells were routinely cultured in standard growth medium (RPMI1640, Sigma-Aldrich, St. Louis, MO) containing 50 μM 2-mercaptoethanol (Sigma-Aldrich, St. Louis, MO), 1 mM sodium pyruvate (Wako, Osaka, Japan), and 20% fetal bovine serum. Rcho-1 cell differentiation was induced by culturing in NCTC-135 culture medium (Sigma-Aldrich, St. Louis, MO) containing 50 μM 2-mercaptoethanol, 1 mM sodium pyruvate, and 1% horse serum. For observation of Rcho-1 cell shape, we used CellMask (Thermo Fisher Scientific, Tokyo, Japan) according to the manufacturer’s protocol.

### Animals

Pregnant female Wistar Hannover rats (12 weeks old) were purchased from CLEA Japan. When a vaginal plug was found, the day was designated as 0 days post coitum (0 dpc). Animals were kept in accordance with international guidelines and Japanese law, and the protocol for this study was approved by the Kanazawa Medical University Animal Care and Use Committee.

### Immunohistological assay and Immunocytochemical assay

Placentas obtained from pregnant rats (12 dpc) were fixed with 4% paraformaldehyde, and 10 μm paraffin sections or Rcho-1 cells fixed with 1% paraformaldehyde were prepared. Immunostaining was performed with a rabbit anti-rat Gal-4 polyclonal antibody (Invitrogen. Carlsbad, CA) and a rabbit anti-rat LC3 polyclonal antibody (Wako, Tokyo, Japan), incubated at 4 °C overnight and developed with the Envision system (DAKO, Carpenteria, CA). Double immunofluorescence staining was performed in combination with a rabbit anti-rat Gal-4 polyclonal antibody (Invitrogen, Carlsbad, CA) and either a mouse anti-rat LC3 monoclonal antibody (MBL, Nagoya, Japan) or a mouse anti-rat Cdx2 monoclonal antibody (Abcam, Cambridge, MA), and then detected with anti-rabbit antibody Alexafluor 555 for Gal-4 and anti-mouse IgG antibody Alexafluor 488 for LC3 and Cdx2. Normal rabbit IgG was used as a negative control. Observation was performed using a confocal microscopy. Immunocytochemical assay was performed using Rcho-1 cells on day 1 after induction of differentiation. And then, cells were fixed and incubated with fluorescence antibodies as described above. The stained cells were observed using a confocal microscopy.

### RNA extraction, conventional PCR

Total RNA from pre- (Day 0) and post-differentiated (Day 1, 5, and 7) Rcho-1 cells was extracted using the RNeasy Kit (Qiagen, Valencia, CA) according to the manufacturer’s protocol. Decidua and fetal tissue from the placentas (12 dpc) were separated surgically under a microscope, then total RNA from the rat placentas, decidua and fetal tissue were extracted as described above. Conventional RT-PCR was performed using the GeneAmp^®^ RNA PCR kit (Applied Biosystems, Foster City, CA) according to the manufacturer’s instructions. The gene-specific primer sets were as follows: Gal-4 (Forward 5′-GTCATGTCTGAGCACTACAAGGTC-3′; Reverse 5′-TCAGATCTGGACATAGGACAA -3′) (657 bp). *Gapdh* mRNA levels were used as an internal standard for calibration (Forward 5′-AAGGTGGTGAAGCAGGC-3′; Reverse 5′-CCCCAGGCCCCTCCTGTTGT-3′) (383 bp).

### DNA microarray analysis

Total RNA from pre- and post-differentiated (day 7) Rcho-1 cells was extracted using the RNeasy Kit (Qiagen, Valencia, CA) according to the manufacturer’s protocol. Preparation of cDNA, target hybridization using the Affymetrix Rat Gene 1.0 ST array (Affymetrix, Santa Clara, CA), and data processing were performed as described in a previous report^37^.

### Quantitative assay

For quantitative assessment, total RNA obtained from three independent experiments was used. The mRNA levels were evaluated by SYBR Green I-based real-time RT-PCR with an ABI PRISM 7000 (Applied Biosystems, Foster City, CA). All gene primer sets were purchased from Takara Bio (Otsu, Japan). *Gapdh* mRNA levels were used as an internal standard for calibration.

### Over-expression of Gal-4 in Rcho-1 cells

The plasmid for over-expression of Gal-4, pEF1α/Gal-4, was constructed as follows. The full length coding cDNA sequence of rat Gal-4 (bases 13 to 987 of GenBank Accession: NM_012975) was inserted into the multiple cloning site of the pEF1α-IRES-ZsGreen1 vector (Clontech, Palo Alto, CA), which is a bicistronic mammalian expression vector that allows the simultaneous, constitutive expression of a protein of interest and the green fluorescent protein ZsGreen1. Rcho-1 cells in the proliferative phase were transfected with pEF1α/Gal-4 to express Gal-4 using Lipofectamine LTX (Thermo Fisher Scientific, Tokyo, Japan). An empty vector was used for control experiments. For cell-size and DNA contents analysis, the cells were induced to differentiate after culturing for an additional 3 days, and cells were harvested at 7 days after induction of differentiation. Cell-size of Rcho-1 cells was analyzed using a FACSCalibur cytometer (BD Biosciences, Franklin Lakes, NJ). DNA contents assay was performed by PI staining and Flow cytometry.

### Time-lapse recording

Rcho-1 cells were cultured in a 6 cm dish (Matsunami, Japan) and pEF1a/Gal-4 or an empty control vector was transfected as described above. Time-lapse recording began after exchanging the medium from RPMI1640 to differentiation medium (NCTC/1% horse serum) at 48 hrs post transfection using a LCV110 microscope (Olympus, Japan) which acquired a z-stack image every 30 minutes, for a total of 12 hrs. All file handling and image analysis was done in ImageJ (http://imagej.nih.gov). The number of adherent ZsGreen-positive cells was counted 12 hrs after time-lapse recording was started. The proportion per unit time of adherent cells was represented as *p*. By use of binomial distribution based on the 4-th trial number and *p*, the proportion of cells in contact with 0–3 ZsGreen-positive cells was hypothesized to be ((1*-p*)^3^, 3(1-*p*)^2^*p*, 3(1-*p*)*p*^2^, *p*^3^) which was derived from the following steps; Step1(1, 0, 0, 0), Step2(1-*p*, *p*, 0, 0), Step3((1-*p*)^2^, 2(1-*p*)*p*, *p*^2^, 0), Step4((1-*p*)^3^, 3(1-*p*)^2^*p*, 3(1-*p*)*p*^2^, *p*^3^). Each *p* of Gal-4 or empty vector was calculated using a least squares method by comparing the experimental data and the components of Step4.

### Zymography assay

Zymography assays were performed using the Gelatin-zymography kit (ATTO type) according to the manufacturer’s protocol (Cosmo Bio, Japan). In brief, Rcho-1 cells were harvested before and after differentiation. Cells were dissolved in cell lysis buffer and fractionated by gel electrophoresis. After washing the gels with wash buffer, MMP activity was determined by the degree of degradation of specific substrates.

### Western blot assay

Harvested cell pellets were dissolved in SDS lysis buffer, boiled, fractionated on an SDS-polyacrylamide gel, and transferred to a nitrocellulose membrane. After blocking with PBS plus 0.1% Tween-20 containing 5% skim milk for 1 hr at room temperature, the membranes were incubated with antibodies against LC3 (Wako, Japan), Gal-4 (Invitrogen, Cambridge, USA) and tubulin (Santa Cruz, Columbia, SC) overnight at 4 °C. After washing with PBS plus 0.1% Tween-20, membranes were incubated with an anti-HRP-linked antibody for 1 hr at room temperature and visualized with Western Lightning Chemiluminescent Reagent (PerkinElmer, Waltham, USA) according to the manufacturer’s protocol.

### Invasion assay

Invasion assay was performed using the 24 well BME-coated Cell Invasion kit (Trevigen, Inc.) according to the manufacturer’s protocol. In brief, Rcho-1 cells induced to differentiate with or without either 3 mM 3-MA or 3 nM Bafilomycin A for 24 hrs were harvested and transferred into the transwells at 1.0 × 10^5^ cells/well. After incubation with each inhibitor for 48 hrs, the invasive cells attached to the lower surface of membrane insert were washed and stained with Calcein-AM for 1 h at 37 °C. The fluorescence was detected by Envision Multilabel Reader (PerkinElmer, Wallac Oy, Finland). The fluorescent value was standardized to a vehicle control.

### Statistical analysis

For statistical comparison, Student’s t-test was used. All statistical analyses were performed with Prism4 software (GraphPad Software,La Jolla, CA).

## Additional Information

**How to cite this article**: Arikawa, T. *et al*. Galectin-4 expression is down-regulated in response to autophagy during differentiation of rat trophoblast cells. *Sci. Rep.* **6**, 32248; doi: 10.1038/srep32248 (2016).

## Supplementary Material

Supplementary Information
