# Supplementary material for: Galectin-4 expression is down-regulated in response to autophagy during differentiation of rat trophoblast cells
Source: Sci Rep. 2016 Aug 30;6:32248. doi: 10.1038/srep32248 (PMC5004202; doi:10.1038/srep32248)
Supplement: Supplementary Information [file srep32248-s1.pdf]

Supplementary Information

Manuscript No: SREP-16-07311A

Galectin-4 expression is down-regulated in response to autophagy during differentiation of rat trophoblast cells

Tomohiro Arikawa, Shengjun Liao, Hiroki Shimada, Tomoki Inoue, Hiromi Sakata-Haga, Takanori Nakamura, Toshihisa Hatta, and Hiroki Shoji

Supplementary Figure Legends

Supplementary Figure S1

Full-length blot of Figure 2A

Supplementary Figure S2

(A) Full-length gel of Figures 4D. (B) Full-length blot of Figure 4E.

Supplementary Figure S3

Full-length blot of Figure 5B

Supplementary Figure S4

(A) Full-length Zymography gel of Figure 6B. (B) Full-length Zymography gel of Figure 6D. (C) Full-length Zymography gel of Figure 6F.

Supplementary
Figure S1

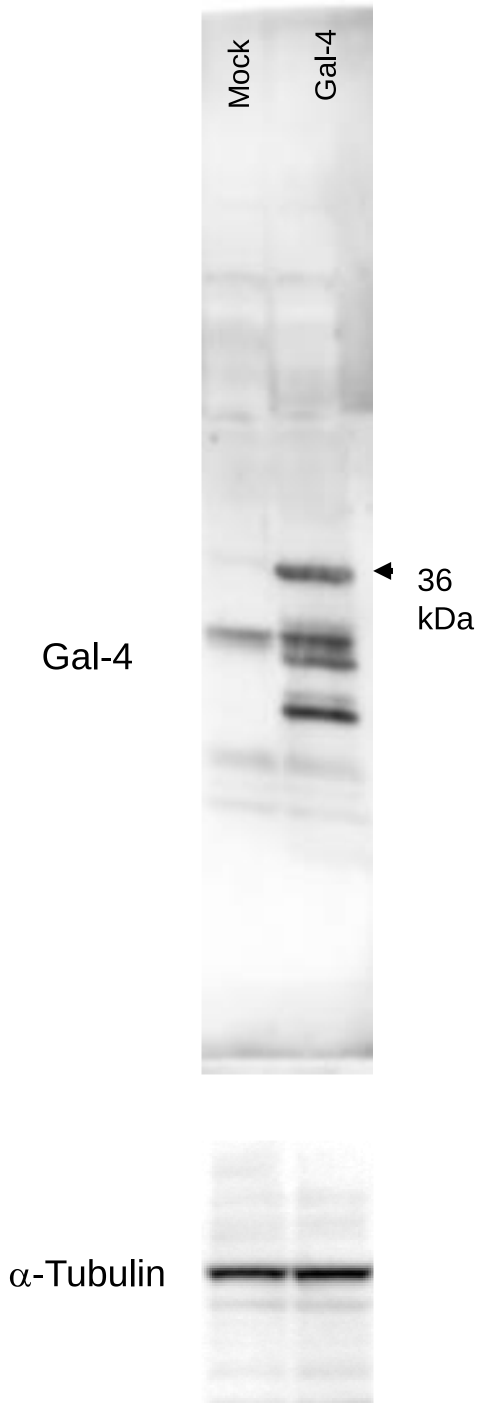

Supplementary Figure S1
Full-length blot of Figure 2A.

Supplementary Figure S2

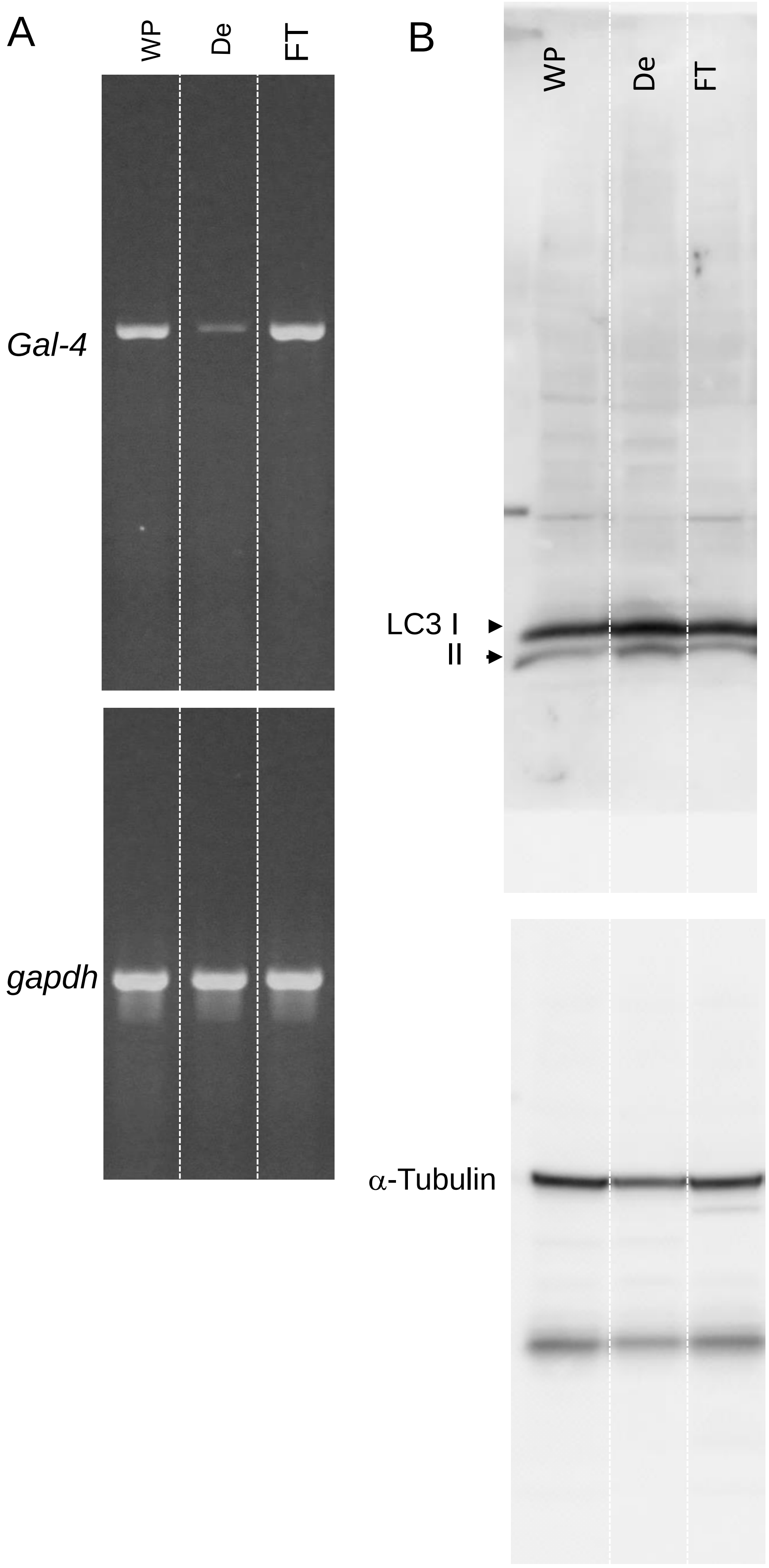

Supplementary Figure S2
(A) Full-length gel of Figures 4D. (B) Full-length blot of Figure 4E.

Supplementary
Figure S3

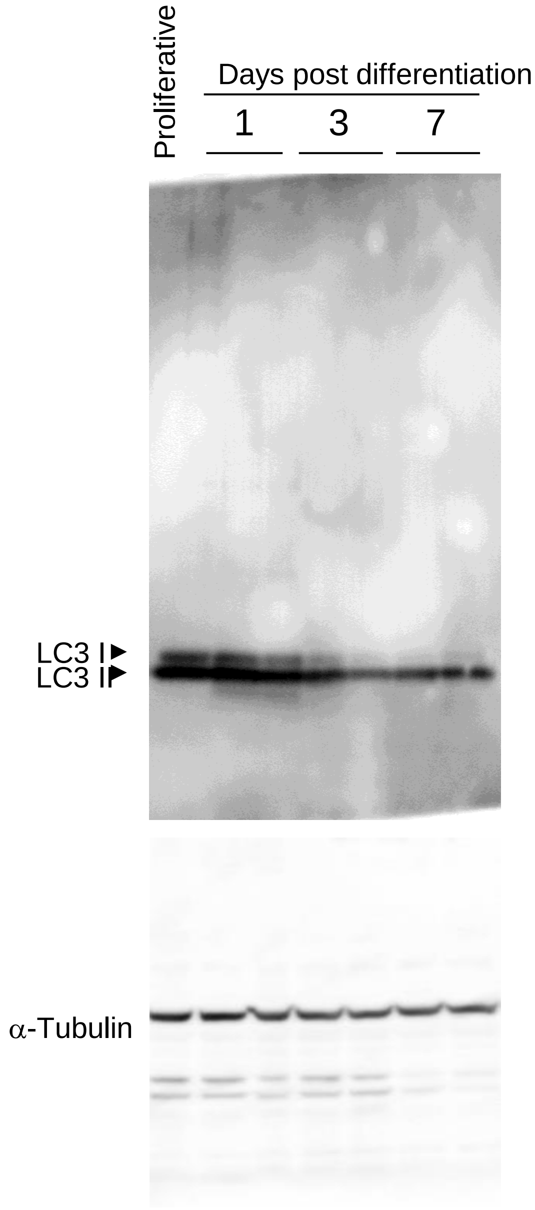

Supplementary Figure S3
Full-length blot of Figure 5B.

Supplementary Figure S4

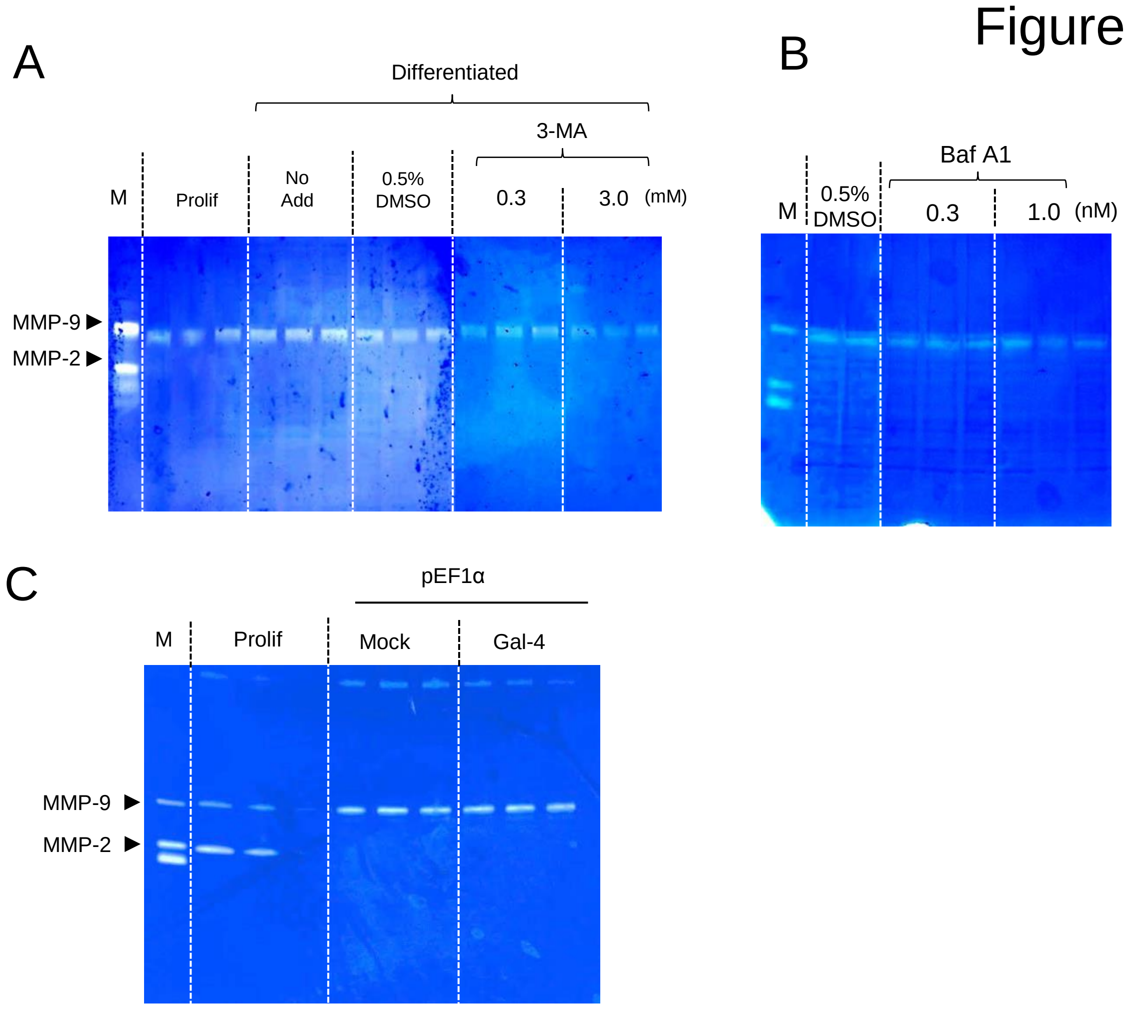

Supplementary Figure S4
(A) Full-length Zymography gel of Figure 6B. (B) Full-length Zymography gel of Figure 6D. (C) Full-length Zymography gel of Figure 6F.
